# Analysis of Threshold Changes of Tumor Mutation Burden of Gastric Cancer and Its Relationship with Patients' Prognosis

**DOI:** 10.1155/2021/9026610

**Published:** 2021-09-27

**Authors:** Nan Zhang, Peiyu Li, Xin Wu, Shaoyou Xia, Xudong Zhao, Lin Chen

**Affiliations:** Department of General Surgery, The First Medical Centre, Chinese PLA General Hospital, Beijing 100036, China

## Abstract

**Objective:**

Gastric cancer is a malignant tumor originating from gastric mucosal epithelium. Here, we aimed to investigate the analysis of the threshold change of gastric cancer tumor mutation burden (TMB) and its relationship with the prognosis of patients.

**Methods:**

256 patients with gastric cancer were selected as subjects. All patients were in the advanced stage and received surgical resection of D2 lymph node dissection. After the operation, a follow-up was performed for 24 months, and the disease-free survival and overall survival of patients were counted. The NGS molecular biological was detected to obtain gastric cancer tumor mutation burden (TMB) data. Pearson correlation analysis software was used to analyze the correlation between TMB threshold and disease-free survival or overall survival of patients with gastric cancer, and the multivariate logistic analysis was performed as well.

**Results:**

The disease-free survival period and the overall survival period of patients in the low-to-medium TMB group were both longer than those in the high TMB group. Pearson correlation analysis results showed that the TMB threshold was negatively correlated with the disease-free survival and overall survival of gastric cancer patients. Results from multivariate logistic analysis showed that high TMB thresholds have a greater impact on disease-free survival and overall survival of patients, but the impact of medium and low TMB thresholds on disease-free survival and overall survival of patients is weakened.

**Conclusions:**

The TMB threshold level has a predictive effect on the effect of surgical resection of D2 lymph node dissection, and high levels of TMB can significantly affect disease-free survival and overall survival of patients with advanced gastric cancer.

## 1. Introduction

Gastric cancer is a malignant tumor that originates from the epithelium of the gastric mucosa, and with the changes in the diet of Chinese residents, increased work pressure, and *Helicobacter pylori* infection, the incidence of the disease is showing a younger trend [1]. Previous studies have shown that gastric cancer is more likely to occur in people over the age of 50, and the incidence of men is slightly higher than that of women, and more than 50.0% of patients occur in the antrum, greater curvature, and lesser curvature of the stomach [2]. Due to the lack of typical clinical symptoms in the early stage of gastric cancer, the diagnosis of most patients is already in the middle and late stages, and the best diagnosis and treatment opportunity are missed [2,3]. Surgical resection of D2 lymph node dissection is a common treatment method for patients with gastric cancer. The lesion tissue can be removed with surgery, but most patients lack effective evaluation methods, resulting in poor long-term prognosis [4,5]. Tumor mutational burden (TMB), an emerging independent biomarker, is the detection of the total number of mutations in the coding region of tumor genes, which is widely used to stratify the patient's response to treatment. Previous studies have shown that TMB refers to the exon coding region of the evaluation gene in the tumor cell genome and the total number of substitution, insertion, or deletion mutations per megabase [6]. Highly mutated tumors are believed to contain increased neoantigen load, making them immunogenic and responsive to immunotherapy [7]. However, there are few studies on the relationship between TMB and the prognosis of patients with gastric cancer.

In this study, gastric cancer patients were selected as subjects to explore the changes of TMB and its relationship with the prognosis of the gastric cancer patients. The report is as follows.

## 2. Materials and Methods

### 2.1. Clinical Data

256 patients with advanced gastric cancer were selected as subjects from May 2017 to January 2019 at the First Medical Centre, Chinese PLA General Hospital, including 185 males and 71 females, aged 45–84 years, with an average of 68.83 ± 5.71 years old. ECOG score: 0 points (149 cases); 1 point (107 cases). Tumor location: 42 cases of upper gastric body, 75 cases of gastric body, and 139 cases of gastric isthmus. Clinical N stage: 34 cases of cN1 stage, 132 cases of cN2 stage, and 90 cases of cN3 stage. The study was approved by the Ethics Committee of the First Medical Centre, Chinese PLA General Hospital, and the informed consent was obtained from the patients.

### 2.2. Inclusion and Exclusion Criteria

Inclusion criteria: (1) the patients met the diagnostic criteria for gastric cancer, were diagnosed by pathological tissues, and were in the advanced stage. (2) All patients were planned to undergo surgical resection of D2 lymph node dissection, and the patients can tolerate it. (3) The complete baseline and follow-up data were available.

Exclusion criteria: (1) the recurrent gastric cancer, patients undergoing emergency surgery due to tumor perforation, obstruction, or bleeding. (2) Mental disorders, abnormal blood coagulation, or autoimmune system diseases. (3) Tumors in other parts or those undergoing radiotherapy and chemotherapy before surgery.

### 2.3. Method

#### 2.3.1. Treatment Method

The specific method of surgical removal of D2 lymph node dissection is as follows. The patient takes the supine position and then undergoes general anesthesia, disinfection, and draping. Laparoscopy and other surgical instruments used to separate the omentum, the anterior lobe of the transverse mesocolon to the tail of the pancreas, separate the pancreatic capsule, clip off the left side of the gastric omentum, and the tip splenogastric ligament. The gastrophrenic ligament was opened, and the dissection retrograded to remove lymph nodes around the splenic artery after sweeping the splenic hilar area. The right omentum was peeled to the liver flexure of the colon, the head of the pancreas and the bulb of the duodenum were fully exposed, the right blood vessels of the gastroomentum was clipped off, and the sixth group of lymph node dissection was completed. After the above operations were completed, the omental sac was incised, the lymph nodes of group 12a were cleaned, the tissue separation was completed along the direction of the common hepatic artery, the intrathecal area was routinely cleaned, and the left gastric vein and left gastric artery were dissected, and the lymph nodes of group 8, 9, and 11p were dissected. The posterior wall of the stomach, the cardia, and the lower esophagus were free, and then, a surgical incision in the lower abdomen of the patient was made to complete the cut of the lower esophagus, the specimen was taken out, and the digestive tract reconstruction was completed. After the operation, a follow-up was performed for 24 months, and the disease-free survival and overall survival of patients were counted [8].

#### 2.3.2. Determination of TMB

After the operation, the NGS molecular biological was performed to obtain the TMB data of gastric cancer. The specific methods are as follows. (1) Specimen processing: gastric cancer tissues were fixed with formaldehyde and embedded in paraffin. 6–10 um sections were prepared, and then, hematoxylin-eosin (H&E) staining was performed, and the tumor cell content was judged by pathologists. The tissues with tumor cell content >10.0% were selected. The plasma samples were collected with EDTA anticoagulation tube, centrifuged for 10 min (1800 rpm, centrifugal radius 15 cm), and then placed at −4°C until use. (2) DNA extraction and library preparation: Qiagen company extraction kit was used to extract tissues and the DNA of white blood cells. The KAPA Hyper Prep Kit (Woosen Biotechnology, Shanghai, China) was used as the sequencing library, and the target gene was enriched after PCR amplification and library purification [9]. (3) Enrichment and sequencing of target genes: after the above operations were completed, multiple samples were mixed to obtain 2 ug of DNA mixed library. The colonized biotin-type DNA probe (GeneSeqOne^TM^) was used to hybridize and capture 415 coding regions of genes related to gastric cancer and 16 introns of genes in the library. Meanwhile, the library DNA was amplified with the help of Illumina p5, p7 primers, and KAPA HiFi HotStart ReadyMix (Sigma-Aldrich, Shanghai, China), and the enriched library was sequenced on the HiSeq 4000 platform using a 2×150 bp sequencing kit [10]. (4) Sequencing data processing: Trimmomatic 25 software was used to filter the sequencing data and remove low-quality bases or N bases. The resulting data and the reference sequence genome were compared through high-throughput sequencing analysis. GATK was used to complete single calculation of mutation and indel gene mutation data. (5) TMB calculation: TMB was defined as the number of tumor-specific mutations contained in the 1 Mb base of the coding region. The data of tumor samples and white blood cells were compared, and the somatic mutations were analyzed and germline mutations were removed [11].

#### 2.3.3. Correlation Analysis

Pearson correlation analysis software was used to analyze the correlation between TMB threshold of gastric cancer patients and disease-free survival and overall survival and multivariate logistic analysis.

### 2.4. Statistical Analysis

SPSS24.0 software (IBM, NY, USA) was used to measure the statistical data. The count data were analyzed by the *χ*^2^ test, expressed by *n* (%). The measurement data in the article were all in accordance with the normal distribution and analyzed by the *t*-test, expressed by ($\bar{x}\pm s$). Kaplan–Meier survival analysis was carried out for overall survival. *P* < 0.05 was considered as statistical significance.

## 3. Results

### 3.1. Comparison of Disease-Free Survival and Overall Survival of Patients with Different TMB Levels in Gastric Cancer

**Figure 1:**
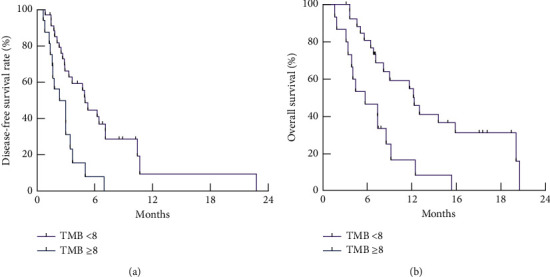
Comparison of disease-free survival and overall survival of patients with different TMB levels in gastric cancer. (a) Kaplan–Meier survival analysis of disease-free survival time of patients with different TMB levels. (b) Kaplan–Meier survival analysis of overall survival time of patients with different TMB levels.

The TMB level in advanced gastric cancer was 5–14, with an average of 8.58 ± 0.61. The patients with gastric cancer were divided into the high TMB group (*n* = 131 cases, TMB≥8) and low-medium TMB group (*n* = 125 cases, TMB<8) by the three-point method. The enrolled patients were followed up for 24 months. Results showed that the disease-free survival time of patients in the low-to-medium TMB group was 14.39 ± 3.23 months, and the overall survival time was 17.41 ± 2.12 months, which were longer than the disease-free survival time (11.51 ± 2.59 months) and the overall survival time of the high TMB group (13.15 ± 2.05 months) (*P* < 0.05; *P* < 0.05) (Figures 1(a) and 1(b)).

### 3.2. Correlation between TMB Threshold and Disease-Free Survival and Overall Survival of Patients with Gastric Cancer

Results from Pearson correlation analysis showed that the TMB threshold of gastric cancer patients was negatively correlated with disease-free survival and overall survival (*P* < 0.05), as given in Table 1.

### 3.3. Multivariate Analysis of TMB Threshold and Disease-Free Survival and Overall Survival of Patients with Gastric Cancer

Results from multivariate logistic analysis showed that the high TMB threshold of gastric cancer patients had a greater impact on disease-free survival and overall survival of patients, but the impact of medium and low TMB thresholds on disease-free survival and overall survival of patients was weakened, as given in Table 2 and Table 3.

## 4. Discussion

In recent years, the number of advanced gastric cancer in China has been increasing, and 60.0%–80.0% of gastric cancer patients are already in the advanced tumor stage [12]. Surgical removal of D2 lymph node dissection is a common surgical treatment method for patients with advanced gastric cancer. However, due to the lack of effective prediction and evaluation indicators during the operation of some patients, disease-free survival and overall survival rate of patients are low, which affects the prognosis of patients. Therefore, effective evaluation indicators are the focus of clinical evaluation and research for gastric cancer.

TMB is currently a hotspot in clinical research. TMB refers to the total number of substitution, insertion, and deletion mutations per megabase in the exon coding region of the evaluated gene in the tumor cell genome [13]. Previous studies have shown that changes in the pathogenicity of cell functions due to changes in the genome are clinically called “driver mutations,” which can lead to tumors [14]. In this study, the level of TMB in advanced gastric cancer was 5–14, with an average of 8.58 ± 0.61. The disease-free survival time of patients in the low-to-medium TMB group was 14.39 ± 3.23 months, and the overall survival period was 17.41 ± 2.12 months, both of which were longer than those in the high TMB group, (11.51 ± 2.59) months and (13.15 ± 2.05) months (*P* < 0.05). These results suggest that the survival and overall survival of patients in the high TMB group are lower. Rizzo and Ricci revealed that TMB has a significant effect on the differential expression of hepatocellular cancer genes and the proportion of infiltrating immune cells in tumor tissues [15]. Although this study is not the same disease as this article, it fully illustrates the correlation between TMB and tumors.

NCCN guidelines included TMB testing for the first time in 2019, which further promoted the clinical application of TMB [16]. Increasing research studies have confirmed that high levels of TMB are correlated with the survival rate of patients with different types of tumors, leading to lower patient survival and disease-free survival [17]. TMB reflects the repair and damage of DNA in tumor cells to a certain extent and is related to the ability to produce tumor neoantigens. DNA mismatch repair (MMR) is responsible for repairing DNA replication errors, and mutations associated with MMR often cause picoinstability. Therefore, hyper-microsatellite instability can be used as a surrogate indicator for MMR functional defects. In order to further analyze the relationship between the TMB threshold and the prognosis of patients with gastric cancer, Pearson correlation analysis was performed in this study. Results showed that the TMB threshold of gastric cancer patients was negatively correlated with disease-free survival and overall survival. Multivariate logistic analysis results showed that the high TMB threshold of gastric cancer patients has a greater impact on disease-free survival and overall survival, but the impact of medium and low TMB thresholds on disease-free survival and overall survival of patients was weakened, indicating the close relationship between the TMB of advanced gastric cancer and the patient's prognosis. Therefore, when surgical treatment of patients with diagnosed advanced gastric cancer is performed clinically, the measurement of the patient's TMB level should be strengthened, the long-term prognosis of the patient should be evaluated, and the corresponding measures should be formulated according to the measurement results to intervene to consolidate the surgical effect and promote the recovery of the patient [18].

This study also has its limitations. First, the relatively small sample size may lead to errors in overall survival. Second, the small number of human and environmental factors may also lead to statistical errors. Third, more biological functions in vitro are required to explore in the future studies.

In summary, for patients with advanced gastric cancer, the tumor mutation load threshold level has a predictive effect on the effect of surgical resection of D2 lymph node dissection, and high levels of TMB could significantly affect the disease-free survival and overall survival of patients, which provided a certain reference value for the evaluation of prognosis in gastric cancer patients.

## Figures and Tables

**Table 1:** Correlation between TMB threshold and disease-free survival and overall survival of patients with gastric cancer (*r*, *P*).

Correlation	Disease-free survival	Overall survival
*r*	−0.715	−0.892
*P*	<0.001	<0.001

**Table 2:** Multivariate analysis of TMB threshold and disease-free survival of patients with gastric cancer.

Multivariate	Β value	S.E	Wald	*P* value	OR value	95% CI
High TMB	1.973	0.121	9.434	<0.001	7.982	6.313–8.493
Medium TMB	1.495	0.104	8.498	<0.001	6.791	6.312–7.326
Low TMB	1.213	0.097	7.151	<0.001	5.457	4.698–7132

**Table 3:** Multivariate analysis of TMB threshold and disease-free survival of patients with gastric cancer.

Multivariate	Β value	S.E	Wald	*P* value	OR value	95% CI
High TMB	1.943	0.133	5.456	<0.001	5.412	4.951–5.838
Medium TMB	1.783	0.214	6.981	<0.001	4.313	3.235–7.491
Low TMB	1.669	0.169	4.341	<0.001	6.323	4.346–7.982

## Data Availability

The datasets used and/or analyzed during the present study are available from the corresponding author upon request.
